# African Swine Fever Virus in Pork Brought into South Korea by Travelers from China, August 2018

**DOI:** 10.3201/eid2506.181684

**Published:** 2019-06

**Authors:** Hyun-Joo Kim, Min-Jung Lee, Soo-Kyoung Lee, Da-young Kim, Sang-Ji Seo, Hae-Eun Kang, Hyang-Mi Nam

**Affiliations:** Animal and Plant Quarantine Agency, Gimcheon, South Korea

**Keywords:** African swine fever virus, food items containing pork, China, pork, viruses, outbreak, food safety, South Korea

## Abstract

We tested samples of pork products confiscated from travelers to South Korea for African swine fever virus (ASFV). We detected ASFV in 4 food items confiscated from travelers from Shenyang, China, in August 2018. Surveillance of pork products at country entry points is needed to mitigate the risk for ASFV introduction.

African swine fever (ASF) is a fatal viral disease that affects pigs of all ages and breeds. ASF virus (ASFV) is highly virulent and remains a global threat because of the lack of a vaccine and the ability of the virus to survive in various environmental conditions. Since 2007, ASFV has been spreading across Europe and Russia. In August 2018, China reported the first outbreak of ASF in Asia (*1*). Since then, ASFV has been reported in numerous provinces and continues to spread across China (*2*).

Although ASF has never occurred in the Republic of Korea (hereafter referred to as South Korea), ASFV could be introduced into this country through various routes. The risk for ASF introduction into South Korea increases with the continuous spread of the disease across China. Pork products contaminated with ASFV are among the main risk factors for spreading the disease (*3*). Hence, since 2015, we have been conducting surveillance on pork products confiscated at airports and ports from travelers coming from countries affected by ASF. Since the program started in 2015, an average of 6,200 products have been seized per month, and we tested an average of 10 (0.16%) products per month.

After the first ASF outbreak in China, South Korea enhanced quarantine inspections of travelers, especially those coming from China. A total of 4,064 pork products (3,374 sausages, 12 hams, and 678 other products containing pork) were seized from travelers from China in August 2018. Among these products, we randomly selected samples from 52 (1.28%) products for real-time PCR testing. We homogenized these samples and extracted nucleic acids using High Pure PCR Template Preparation Kit (Roche, https://www.roche.com) in a Biosafety Level 3 laboratory at the Animal and Plant Quarantine Agency in Gimcheon, South Korea. We used ASFV OURT88/3 virus as a positive control. To amplify the ASFV *B646L* gene, we performed TaqMan real-time PCR (Applied Biosystems, https://www.thermofisher.com) as recommended (*4*).

In total, 4 samples from China tested positive for ASFV: 2 blood sausages (identification [ID] no. 18083111, seized August 16, 2018, and ID no. 18081148, seized August 20, 2018), 1 dumpling (ID no. 18082721, seized August 18, 2018), and 1 commercial sausage product (ID no. JI 18080406, seized August 26, 2018). All ASFV-positive samples were from products seized at the Incheon and Jeju International Airports from passengers flying from Shenyang, China, where the first ASFV outbreak in China was reported.

We performed conventional PCR to further analyze the ASFV isolates detected. We amplified 3 independent regions of the ASFV genome: the *B646L* gene encoding p72, the *E183L* gene encoding p54, and a tandem-repeat sequence located between the *I73R* and *I329L* genes (*5*–*7*). All genes detected were ASFV genotype II (Figure). All positive samples had an intergenic region II variant with an additional tandem-repeat sequence (5′-GGAATATATA-3′) between the *I73R* and *I329L* genes (*5*). The intergenic region II variant we observed was identical to those reported in isolates Ukr12/Zapo, Bel13/Grodno, Lt14/1490, Lt14/1482, Pol14/Sz, and Pol14/Krus (*6*). The same tandem-repeat sequence insertion was also observed in China isolates ASFV SY18 and CN201801 (*1**,**2*).

**Figure:**
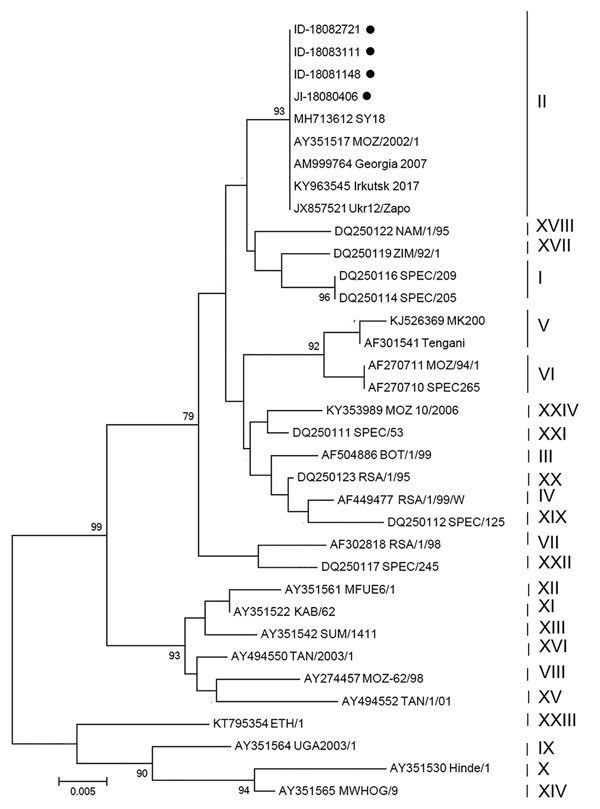
Phylogenic analysis of partial *B646L* gene sequence of African swine fever virus (ASFV) in samples of pork products brought into South Korea by travelers from Shunyang, China, August 2018, and reference sequences. Neighbor-joining phylogenic tree was constructed by using MEGA 6.0 (https://www.megasoftware.net). Black dots indicate genes of ASFV isolates detected in 3 food items containing pork and 1 commercial pork product confiscated from travelers. Vertical lines at right indicate ASFV genotypes I–XXIV. GenBank accession numbers are provided for reference sequences. Scale bar indicates the number of base pair substitutions per nucleotide.

We compared the gene sequences of our samples with those of ASFV SY18 (GenBank accession nos. MH713612, MH717102, and MH717104). The genes detected in the blood sausage samples (ID nos. 18083111 and 18082721) were 100% identical to those of ASFV SY18. However, the *E183L* gene and *I73R*–*I329L* intergenic spacer were not detectable in the other 2 samples (ID nos. 18081148 and JI 18080406), suggesting that only part of the ASFV genome remained intact and preventing more thorough genomic comparisons. We also attempted to culture virus from the 4 samples in primary pig alveolar macrophage cells using the procedure established by the Center for Animal Health Research (http://asf-referencelab.info/asf/en/procedures-diagnosis/sops), the European Union Reference Laboratory for ASF in Madrid, Spain, but the virus did not propagate.

Our results indicate that the ASFV isolates detected in South Korea in August 2018 were identical to those reported in China. Also, the detection of ASFV genes in commercial sausage products from China indicates that pigs infected with ASFV might have been slaughtered and distributed in markets throughout China.

The Food and Agriculture Organization of the United Nations reported the possibility of ASFV spread from China to other parts of Asia, including the Korean Peninsula and Japan (*8*). Table and kitchen scraps containing pork contaminated with ASFV was reported to be a potential source of the infection in China (*9*). Contaminated pork products fed to pigs have often been the source of ASFV outbreaks and introduction into previously unaffected areas (*10*). Thus, detection of ASFV in these confiscated products highlights the importance of surveillance at points of entry to mitigate the risk for ASFV introduction through illegal imports.
